# Maximum thickness of non-buffer limited electro-active biofilms decreases at higher anode potentials

**DOI:** 10.1016/j.bioflm.2022.100092

**Published:** 2022-11-11

**Authors:** João Pereira, Guanxiong Wang, Tom Sleutels, Bert Hamelers, Annemiek ter Heijne

**Affiliations:** a Wetsus, European Centre of Excellence for Sustainable Water Technology, Oostergoweg 9, 8911MA, Leeuwarden, the Netherlands; b Environmental Technology, Wageningen University, Bornse Weilanden 9, P.O. Box 17, 6700 AA, Wageningen, the Netherlands; c Faculty of Science and Engineering, University of Groningen, Nijenborgh 4, 9747 AG Groningen, the Netherlands

**Keywords:** Electro-active biofilms, Buffer limitation, Maximum thickness, Identification model

## Abstract

The accumulation of protons in electro-active biofilms (EABfs) has been reported as a critical parameter determining produced currents at the anode since the very beginning of the studies on Bio-electrochemical systems (BESs). Even though the knowledge gained on the influence of this parameter on the produced currents, its influence on EABfs growth is frequently overlooked. In this study, we quantified EABfs thicknesses in real-time and related them to the produced current at three buffer concentrations, two anode potentials and two acetate concentrations. The thickest EABfs (80 μm) and higher produced currents (2.5 A.m^−2^) were measured when a 50 mM buffer concentration was used. By combining the measured EABfs thicknesses with the pH in the anolyte, a simple model was developed to identify buffer limitations. Buffer limited EABfs with thicknesses of 15 and 42 μm were identified at −0.3 V vs Ag/AgCl when 10 and 50 mM buffer concentrations were used, respectively. At −0.2 V vs Ag/AgCl, the thicknesses of buffer limited EABfs decreased to 13 and 20 μm, respectively. The model also estimated buffer and acetate diffusion rates in EABfs and allowed to determine the boundary between a buffer and acetate limited EABfs. The diffusion rates reported in this study and the definition of the boundary between buffer and acetate limited EABfs provide a powerful tool to avoid limitations, leading to higher produced currents at the anode.

## Highlights

• The effect of anolyte buffer concentration on the thickness of EABfs was studied.
• Maximum thickness of 78 μm was measured for EABfs buffered with 50 mM.
• Anolyte pH was used to determine buffer limitations.
• More positive anode potential decreased thickness of non-buffer limited EABfs.
• Boundary between acetate and buffer limited EABfs was determined.

## 1 Introduction

Bio-electrochemical systems (BESs) combine electro-active bacteria and electrodes [1,2]. These bacteria are the catalysts and the conductive biomaterial between organic and electrical energy. When growing on an anode, these bacteria catabolize chemical compounds such as acetate molecules and generate electrical energy by using the anode as final electron acceptor [3]. Besides allowing to recover electrical energy in an external electrical circuit, the exchange of electrons with the electrode yields energy for metabolism and the growth of electro-active bacteria on the electrode surface [[4], [5], [6]].

The growth of bacteria on a surface typically leads to the development of a bacterial layer, a so-called biofilm [7]. When combining electro-active bacteria and a solid electrode surface, an electro-active biofilm (EABf) is formed. The growth and thickness of EABfs on an anode depends on several parameters such as the anode material, anode potential and substrate concentration [[8], [9], [10], [11]]. EABfs being the biocatalysts between electron donor and electron acceptor, the produced current at the anode (i.e. reduction of the electron acceptor) is a measure of the activity of EABfs. However, this activity changes as thicker EABfs develop on the anode surface [12].

When thin acetate-fed EABfs grow on the anode, both a complete access to acetate (electron donor) over all the bacterial layers and a contribution of the EABf as a whole to produced current are expected [13]. However, mass transfer limitations are expected when thick EABfs develop on the anode, which result in gradients in the EABf. These gradients in the EABf can be categorized in three main parameters: 1) anode potential, 2) acetate and 3) buffer concentration.

The anode potential and the type of substrate determine the energy bacteria gain when exchanging electrons with the anode [14]. Therefore, the difference between the reduction reaction (meaning the use of the anode as final electron acceptor) and the biological oxidation of the electron donor (approximately −0.5 V vs Ag/AgCl for acetate [15]) is a measure of the available energy gain by bacteria. However, the energy gain is not homogeneous over the whole EABf thickness as the potential of the final electron acceptor decreases as the distance to the anode surface increases [16]. Therefore, due to the lower potential of the redox compounds present in the matrix of the EABfs when compared to the anode surface, the energy gain for the bacteria on the top layers of the EABf decreases. Consequently, even though the top layers of the biofilm are less likely to be deprived in electron donor, the rate of electrons transfer with the anode surface is lower, resulting in lower overall current densities. As opposed to the top layer of the EABf, bacteria present at the anode interface gain more energy by using the anode as electron acceptor but may have limited access to electron donor when the EABf grows too thick. It is therefore important to monitor and control the thickness of EABfs on the anode to circumvent the present of these gradients and to guarantee that EABfs activity is evenly distributed in every layer through the whole EABf thickness on an anode.

The effect the anode potential, acetate concentration, and EABfs thickness on the produced current at the anode has recently been studied [17]. In this study, an increasing current density was observed for EABfs thicknesses up to 40 μm. For thicker EABfs, the overall produced current was constant or even decreased. In the same study, acetate limitations were identified as a reason for constant or decreasing currents, and a maximum thickness of 55 μm was found to sustain non-acetate limited EABfs when the anode potential was controlled at −0.2 V vs Ag/AgCl. Even though the relation between acetate concentration, produced current, and EABfs thickness allowed to identify maximum thicknesses that guarantee non-acetate limited EABfs at different anode potentials, the buffer concentration and eventual buffer limitations as a function of the EABfs thickness were not considered.

The effect of buffer concentration on the produced current by EABfs has thoroughly been explored, and the diffusion of protons (resulting from acetate consumption) out of EABfs has been pointed out as a bottleneck since a very early stage of the studies in BESs [18,19]. These studies emphasize the importance of buffering EABfs and repeatedly indicate that buffer limitations determine the overall activity of EABfs. However, the EABfs thicknesses at which these buffer limitations occur and how these vary as a function of other parameters such as anode potential and acetate concentrations are rarely specified.

Buffer limitation in EABfs is intrinsically related to acetate consumption. When more acetate is consumed, more electrons as well as more protons are generated (ratio of 1 mol of acetate to 8 mol of electron and protons, as described in Equation (1) [20]).

(Eq. 1) CH_3_COO^-^ + 3H_2_O → CO_2_ + HCO_3_^-^ + 8H^+^ + 8 e^-^

**Fig. 1:**
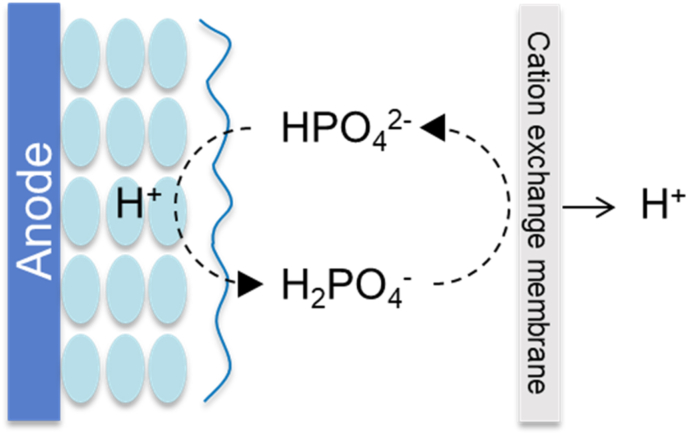
Mechanism of proton association and dissociation using phosphate buffer to remove protons out of the EABf: hydrogen phosphate diffuses in the EABf and takes up one proton forming phosphoric acid; phosphoric acid leaves the EABf and regenerates hydrogen phosphate by transporting the proton though a cation exchange membrane to the cathode.

As previously explained for the acetate diffusion inside EABfs, proton accumulation in EABfs due to limited buffer diffusion inside EABfs cannot be neglected, especially when thick EABfs develop on the anode surface. As a consequence of a pour diffusion of protons out of the EABf, the local pH decreases, and an acidic environment is created. This pH drop decreases the activity of EABfs as less energy can be gained by bacteria (the potential for acetate oxidation increases about 60 mV per pH unit [21]), leading to lower produced currents. To circumvent the accumulation of protons, EABfs are typically buffered to help removing the protons out of the EABf [[22], [23], [24]]. In the case of phosphate buffer, the mechanism involves the penetration of hydrogen phosphate (HPO_4_^2−^) in the EABf, its reduction to phosphoric acid (H_2_PO_4_^−^) taking up one proton, and the regeneration of hydrogen phosphate (the conjugate base) outside the EABf (Fig. 1).

When only considering buffer diffusion driven by concentration gradients, a higher buffering capacity (meaning more hydrogen phosphate diffusion inside the EABfs) is expected by the use of higher buffer concentrations. However, when the EABf grows thick, the rate of hydrogen phosphate diffusion inside the EABfs decreases, and protons accumulation at the bottom layers of the EABf occur. Therefore, measuring the EABf thickness and relate it with the penetration depth of hydrogen phosphate is crucial to identify buffer limitations in EABfs. Besides, even though higher buffer gradients between the inside and outside of EABfs increases the buffer diffusion inside EABfs, the buffering needed to prevent buffer limitations in EABf also depends on the number of protons accumulated in the EABf, which is intrinsically linked to the acetate consumed. Therefore, studying the relation between buffer penetration in EABfs as a function of EABf thickness at different acetate concentrations and anode potentials allows to determine a more accurate maximum thickness of non-buffer limited EABfs and to understand how this maximum thickness changes at different conditions. Besides, integrating acetate and buffer diffusion inside EABfs at different anode potentials also allows to distinguish and define which parameter becomes limiting, and therefore, allowing to identify the boundary between acetate or buffer limited EABfs.

In this study, we aim at determining the effect of buffer concentration on the thickness and produced current by EABfs on an anode. The EABfs were buffered with three different phosphate buffer concentrations (10, 50 and 100 mM) and the EABf thickness was monitored in real-time with Optical Coherence Tomography (OCT). The occurrence of buffer limited EABfs was studied at two non-limiting acetate concentrations (5 and 10 mM) and two anode potentials (−0.2 and −0.3 V vs Ag/AgCl). By calculating the penetration depth of acetate and buffer in the EABfs, maximum thicknesses of non-buffer limited EABfs were estimated. Besides, acetate and buffer diffusion rates in EABfs and specific acetate utilization rate were estimated at two anode potentials and used to determine the boundary between acetate and buffer limited EABfs.

## 2 Material and methods

### 2.1 Experimental setup and reactor design

The electrochemical reactors used in this study have previously been described by Pereira et al., 2022. These reactors were assembled with two equally sized compartments (anode and cathode, each with a volume of 33 cm^3^) separated with a cation exchange membrane (CEM) (Ralex CMHPP, MEGA a.s., Czech Republic). A cation exchange membrane was used to allow the regeneration of the phosphoric acid (acid conjugate of the phosphate buffer) by transporting protons to the cathode, and to avoid the diffusion of hydroxide groups form the cathode into the anode compartment. This way, the effect of the different buffer concentrations on the pH in the anode compartment could be studied. The anode electrode was built with a transparent Fluorine-doped Tin Oxide (FTO) coated glass and a layer of graphite sheet. The FTO electrode had an operating area of 22.3 cm^2^, and the graphite sheet was placed in contact with the FTO electrode and used as current collector. A flat platinum/iridium coated titanium plate (Pt/IrO_2_ 80:20, Magneto special anodes BV, Schiedam, The Netherlands) was placed in the cathode compartment and used as counter electrode.

The anode compartment was operated in continuous mode (at a rate of 0.16 mL min^−1^ and a hydraulic retention time of 23 h) and the cathode compartment in batch mode (i.e. without inflow nor outflow). Both electrolytes, namely anolyte in the anode compartment and catholyte in the cathode compartment, were continuously recirculated at 60 mL min^−1^ (Masterflex L/S, Cole-Parmer, Barendrecht, The Netherlands). The reactors were anode potential controlled by means of a potentiostat (N-stat d-module, Ivium Technologies, Eindhoven, The Netherlands), and the current produced was recorded every minute. The anode potential was measured with an Ag/AgCl reference electrode (+0.203 V vs. Standard Hydrogen Electrode; Prosense, Oosterhout, The Netherlands) that was connected to a Haber–Luggin capillary filled with 3 M KCl and placed in the anode compartment (between the FTO electrode and the CEM). The electrochemical reactors were operated at 298 K in a temperature-controlled cabinet.

### 2.2 Inoculum and electrolyte composition

The inoculum used in these experiments was a mixed culture of active EABfs harvested from acetate-fed anodes. The influent fed into the anode compartment was adapted from the DSMZ culture medium 141 and it constituted of (g.L^−1^): 0.41 and 0.82 NaCH_3_COO, 0.1 MgSO_4_.7H_2_O, 0.74 KCl, 0.58 NaCl, 0.28 NH_4_Cl, 0.1 CaCl_2_.2H_2_O, 1 mL of trace metals mixture and 1 mL of vitamins mixture [25]. This influent was buffered with three different phosphate buffer concentrations (g.L^−1^): 1) 0.68 KH_2_PO_4_ and 0.87 K_2_HPO_4_, 2) 3.40 KH_2_PO_4_ and 4.35 K_2_HPO_4_, and 3) 6.80 KH_2_PO_4_ and 8.70 K_2_HPO_4_. To guarantee the presence of an EABf on the anode, sodium 2-bromoethanesulfonate (2-BES, 1.97 g.L^−1^) was added to the influent to avoid methane formation, and the influent was continuously sparged with nitrogen (before and during the experiments) to keep anaerobic conditions in the anode compartment. The cathode compartment was filled with 50 mM phosphate buffer solution at pH 7, and it was also continuously sparged with nitrogen to avoid accumulation of hydrogen and possible transport through the CEM into the anode. Even though the different phosphate buffer concentrations used in the anolyte and catholyte, no significant buffer leakages over the CEM were measured.

### 2.3 Experimental strategy

This study aimed at understanding the influence of buffer concentration on the performance and growth of EABfs on a FTO electrode. Each experimental runs lasted at least 7 days and all the conditions were tested in duplicate. This dataset contains 128 data points, and all of these are presented to allow depicting trends and to provide the modeling with more information. The anode potentials (−0.2 and −0.3 V vs Ag/AgCl) were chosen based on the results previously reported by Pereira et al., 2022 and aimed at providing enough energy for the development of a wide range of EABfs thicknesses on the FTO electrode (and to be in the range of a reasonable voltage efficiencies when operating the system as a Microbial Fuel Cell). The non-limiting acetate concentrations (5 and 10 mM) were chosen to allow EABf growth and to avoid acetate limited EABfs at a very thin range of thicknesses. Therefore, by allowing the growth of a wide range of EABfs thicknesses and providing non-limited acetate concentration, the study on the effect of the buffer concentration was facilitated.

In total, 12 experimental conditions were tested (two anode potentials, two acetate concentrations and three buffer concentrations) in duplicate. All the 24 experiments are grouped by anode potential in the figures presented in the Result and Discussion section. For each experiment, samples were taken every two or three days after a positive current was observed. Thus, 24 EABfs were grown and their thicknesses were measured over time. Since the focus of the study was to understand buffer limitations in EABfs, the time variable was not included in the axis of the figures presented in the Result and Discussion section.

### 2.4 Acetate consumption, anolyte pH and in-situ monitoring of EABfs thickness

Samples from the anolyte were taken every two or three days throughout each experiment and analyzed to monitor the acetate concentration and pH. The anolyte was initially filtered through a 0.45 μm pore-size filter (EMD Millipore SLFH025NS, Barendrecht, The Netherlands) and the acetate concentration was measured using Ultra-High-Performance Liquid Chromatography (UHPLC) (300 × 7.8 mm Phenomenex Rezex Organic Acid H+ column, Dionex ultimate 3000RS, Thermo Fisher Scientific, The Netherlands). The same sample has used to measure the anolyte pH with a pH electrode (InLab Expert Pro-ISM, Mettler Toledo, USA). The acetate consumed by each EABf (*Ac*_*consumed*_, mol) was calculated as expressed in Equation (2), in which *Ac*_*in*_ (mM) is the acetate concentration in the influent (measured as previously described), *Ac*_*out*_ (mM) is the acetate concentration in the anolyte, *flow* is the flowrate (mL.min^−1^) and *Δt* (min) is the time between samples.

(Eq. 2) *Ac*_*consumed*_ = (*Ac*_*in*_ – *Ac*_*out*_) × *flow* × *Δt*

The monitoring of the thickness of EABfs over time was performed with OCT. For this purpose, the reactors were equipped with Quick-Coupler valves (Swagelok SS-QC4-D-400, USA) to avoid oxygen penetration in the reactors when hydraulically disconnected for the sampling in the OCT. The methodology used here to monitor the growth of EABfs in real time on the FTO electrode has previously been reported [26]. OCT is a visualization technique that allows a non-invasive measurement of biofilm thickness over time. By using near infrared light and by analyzing its scattering, this technique allows imaging biofilm amount and morphology in a micrometer resolution. Briefly, a truthful imaging of the amount of the EABf was obtained by evenly scanning the FTO electrode (in 54 scanning spots) in a procedure that took approximately 45 min. Besides allowing an accurate imaging of EABfs, this duration resulted in no significant changes in the produced current nor acetate concentration in the anolyte before and after sampling in the OCT. The OCT scans were the input for a MATLAB script that isolated and counted the number of pixels representing EABf. These number of pixels were then converted to biomass weight (mg COD) using the calibration line reported [26]. The thickness of the EABfs was calculated by dividing the average volume of each EABf by the area of the electrode (22.3 cm^2^). Given the different anode potentials and acetate concentrations tested here when compared to the conditions previously described by Molenaar et al., 2018, the applicability of the reported calibration line for these experiments was confirmed by measuring the COD of the EABfs at the last day of the experiments. The sampling in the OCT was performed after the anolyte samples to measure the acetate concentration and the anolyte pH were taken (therefore, with the same sampling frequency of two or three days).

### 2.5 Identification of buffer limited EABfs using real time monitoring of EABf thickness and anolyte pH

The accumulation of protons derived from acetate consumption results in decreasing pHs inside EABfs and decreasing produced currents at the anode. The higher the buffer concentration in the anolyte, the higher diffusion of buffer inside the EABf inner layers, and the more efficient diffusion of protons out of the EABfs. A basic model was developed to identify buffer limitations in EABfs by calculating the penetration depth of buffer in EABfs. The penetration depth was calculated using Fick's law (Equation (3)), where *L*_*buffer*_ is the buffer penetration depth (m), *D*_*buffer*_ is the diffusion of buffer (hydrogen phosphate) in the biofilm (m^2^.s^−1^), *HPO*_*4*_^*2−*^ is the concentration of hydrogen phosphate in the anolyte (mol equivalent Ac.m^−3^) and, *k*_*0*_ is the specific acetate utilization rate (mol Ac.m^−3^.s^−1^).

(Eq. 3) *L*_*buffer*_ = √ [(*2* × *D*_*buffer*_ × *HPO*_*4*_^*2−*^)/*k*_*0*_]

The concentration of hydrogen phosphate in the anolyte was chosen to calculate the penetration of buffer inside the EABf as this is the conjugate base that is available to take up one proton and diffuse it out of the EABf. However, the concentration of the hydrogen phosphate in the anolyte is not always equal to the initial concentration added to the influent. The decrease in the available hydrogen phosphate in the anolyte is related to an incomplete regeneration of the hydrogen phosphate (exchange of protons by the phosphoric acid at the CEM), that could be related to a favored transport of other cations though the CEM over protons, or to a pour diffusion of phosphoric acid out of the EABf (however, the diffusion of phosphoric acid is commonly reported to be higher than the diffusion of hydrogen phosphate [18]). As a consequence, the ratio between phosphoric acid and hydrogen phosphate in the anolyte increases, which leads to a decrease in the anolyte pH. Therefore, the concentration of the available hydrogen phosphate based on the anolyte pH was used for a more accurate calculation of the buffer penetration depth in the EABf. This concentration was calculated as described in Equation (4), in which *HPO*_*4*_^*2−*^ is the concentration of hydrogen phosphate in the anolyte (mol equivalent Ac.m^−3^), *anolyte* pH is the measured anolyte pH, *pKa* (7.2) is the pH of the proton dissociation from phosphoric acid to hydrogen phosphate, *Buffer* (mol.m^−3^) is the total buffer concentration added in the influent (both the acid and base conjugates), and *8* is the number of protons generated per mol of acetate consumed.

(Eq. 4) *HPO*_*4*_^*2−*^ = (*10*^*anolyte pH - pKa*^)/ (*1 + 10*^*anolyte pH - pKa*^) x *Buffer*/*8*

Even though the acetate concentrations used in this study aimed at preventing acetate limitations, Fick's law was also used to calculate the penetration depth of acetate inside the EABfs and to compare it to the buffer penetration depth. The acetate penetration depth, *L*_*acetate*_ (m) was calculated using Equation (5), in which *D*_*acetate*_ is the diffusion of acetate in the biofilm (m^2^.s^−1^), and *Ac*_*out*_ is the concentration of acetate in the anolyte (mol Ac.m^−3^).

(Eq. 5) *L*_*acetate*_ = √ [(2 × *D*_*acetate*_ × *Ac*_*out*_)/*k*_*0*_]

Three inputs were used for the model: anolyte pH (to calculate buffer penetration depths), acetate concentration in the anolyte (to calculate acetate penetration depths) and the measured EABfs thicknesses. By using these data, the model estimated acetate and buffer diffusion rates, and specific acetate utilization rate. The specific acetate utilization rate was used to calculate an estimated current density based on the minimum non-limited EABf thickness as described in Equation (6). In this equation, *j*_*estimated*_ represents the estimated current density (mol Ac.m^−2^.s^−1^) and *L*_minimum_ is the minimum EABf thickness, which is the lowest thickness when comparing the calculated buffer penetration depth, acetate penetration depth and the measured EABf thickness.

(Eq. 6) *j*_*estimated*_ = *L*_minimum_ × *k*_*0*_

The calculation of the estimated current density as described above assumes the presence of a fully EABf on the FTO electrode. This is proven by the high range of Coulombic efficiencies (above 95%) obtained in all experimental runs and supported by the inhibition of methanogenesis (with 2-BES) and the absence of H_2_ oxidation in the anode (given the continuous nitrogen sparging in the cathode compartment) (Section A, Appendices).

To merge into a single and realistic estimation of acetate and buffer diffusion rates and specific acetate utilization rates, a minimizing function was chosen to decrease the sum of squares between the estimated and measured current densities. All the 128 data points were used for the modeling approach, and the data were grouped in the two anode potentials used (−0.2 and −0.3 V vs Ag/AgCl). These two anode potentials were assumed constant over the whole measured thicknesses of the EABfs, being the eventual presence of anode potential gradients neglected in the modeling approach. For each anode potential, acetate and buffer diffusion rates and a specific acetate utilization rate are described. The scrip and more details on the model can be found in the Appendices (Section B).

## 3 Results and discussion

### 3.1 Decreasing anolyte pH with increasing currents indicates buffer limitations

**Fig. 2:**
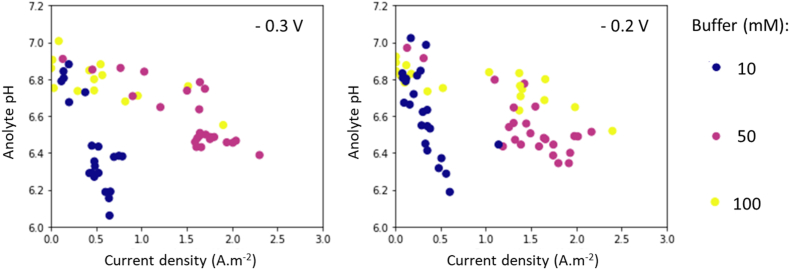
Decrease in the anolyte pH as a function of the current density for the three buffer concentrations (10 mM – blue; 50 mM purple, 100 mM – yellow), when the anode potential was controlled at −0.3 V (left) and −0.2 V (right) vs Ag/AgCl. The steepest decrease was obtained at −0.3 V when 10 mM buffer used, and more stable anolyte pHs were obtained when the EABfs were buffered with 50 and 100 mM. (For interpretation of the references to colour in this figure legend, the reader is referred to the Web version of this article.)

Fig. 2 shows the decrease in the measured anolyte pH as a function of the current density for the different buffer concentrations and anode potentials tested. The decrease in the anolyte pH was steeper for EABfs buffered with 10 mM when compared to higher buffer concentrations. This acidification of the anolyte pH to approximately 6.2, indicating a decrease in the hydrogen phosphate concentration in the anolyte, was observed at a low range of current densities (up to 0.8 A.m^−2^).

Decreasing anolyte pHs were also observed when 50 and 100 mM buffer concentrations were used. However, these decreases were obtained at a higher range of current densities. For both 50 and 100 mM buffer concentration, the anolyte pH fluctuated between 6.6 and 6.9 with increasing current densities up to 1.5 A.m^−2^, and anolyte pHs of approximately 6.4 were measured when the current density further increased up to 2.5 A.m^−2^. At this higher range of current densities, the lowest anolyte pHs were measured when 50 mM buffer concentration was used. A better buffering capacity was thus obtained when 100 mM buffer concentration was used, as the pH in the anolyte kept more stable at higher current densities.

Besides showing that current densities of 2.5 A.m^−2^ were only obtained when the EABfs were buffered with 50 and 100 mM, Fig. 2 also shows that the anode potential had very little effect on the anolyte pH. However, more positive anode potential resulted in higher current densities as it is indicated by the density of data points observed between current densities of 1.0 and 2.5 A.m^−2^ at −0.2 V. The range of current densities obtained and the higher current densities with increasing anode potential are in accordance with previous reported works with EABfs on a flat FTO electrode [17,26,27].

For all the buffer concentrations tested, the observed decrease in anolyte pH as the current density increased indicates an insufficient buffering of the EABfs. However, based on the range of current densities obtained in this study, the anolyte pH would decrease down to 3–4 if no buffer was present in the anolyte (Equation C1, Appendices). The difference between the measured anolyte pH and the estimated anolyte pH in the absence of buffer and the decreasing anolyte pHs with increasing current densities show that EABfs were partially buffered, suggesting buffer limitations.

### 3.2 Thickest EABfs were measured when a buffer concentration of 50 mM was used

**Fig. 3:**
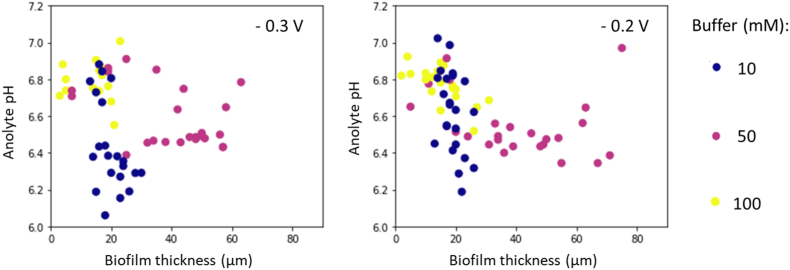
Changes in the anolyte pH as a function of the measured EABf thicknesses for the three buffer concentrations (10 mM – blue; 50 mM purple, 100 mM – yellow), when the anode potential was controlled at −0.3 V (left) and −0.2 V (right) vs Ag/AgCl. Thickest EABfs were obtained with 50 mM buffer concentration. (For interpretation of the references to colour in this figure legend, the reader is referred to the Web version of this article.)

By monitoring the thickness of EABfs in real time with OCT, the effect of the buffer concentration and the anolyte pH on the growth of EABfs can be assessed. Overall, the anolyte pH decreases with increasing EABfs thicknesses (Fig. 3). A maximum EABf thickness of 78 μm was measured when 50 mM buffer concentration and an anode potential of −0.2 V were used. In fact, the widest ranges of EABfs thicknesses were obtained for both applied anode potentials when 50 mM buffer concentration was used. With this buffer concentration, the anolyte pH varied between 6.6 and 6.9 for EABfs thicknesses up to approximately 20 μm (early stages of the EABf development on the anode, with increasing current densities up to 1.5 A.m^−2^), and it decreased to approximately 6.4 when EABfs grew thicker (current densities between 1.5 and 2.5 A.m^−2^). Some anolyte pHs in the range of the 6.6 and 6.9 measured when the EABfs were thicker than 20 μm can be explained by a decrease in current density and/or due to partial EABf detachment from the FTO electrode, both resulting in a slight increase in the anolyte pH. As a consequence, EABf detachment from the FTO electrode, different measured anolyte pHs are shown at a given EABf thickness. Even though equal EABfs thicknesses were measured when part of a thick EABf was washed out and when the EABf was still growing on the FTO electrode, the activity (or current produced by EABfs thickness) of the EABf may differ. Therefore, for the same EABf thickness, when less (or more) current is produced, less (or more) protons have to diffuse out of the EABf, resulting in a higher (or lower) anolyte pH.

The thinnest EABfs were measured when 10 and 100 mM buffer concentrations were used. For the 10 mM buffer concentration, a maximum EABf thickness of approximately 30 μm was measured at both anode potentials. The little growth of EABfs with this low buffer concentration is a consequence of the 1) low current densities (a maximum of 0.8 A.m^−2^) and 2) steep anolyte pH decrease (insufficient buffering). For 100 mM buffer concentration, a similar range of maximum EABfs thicknesses was measured when compared to the 10 mM buffer concentration: approximately 20 μm at −0.3 V and 30 μm at −0.2 V. The thicker EABfs measured at −0.2 V corroborates with the higher current densities measured at this anode potential. Surprisingly, these EABfs did not grow thicker than the EABfs buffered with 50 mM. This little EABf growth is related to the quick decrease in current density after reaching the peak current observed for the EABfs buffered with 100 mM (Section D, Appendices). Even though this decrease to very low current densities cannot be explained with the data collected in this study, these results suggest a negative effect of high buffer concentration in the anolyte on EABfs growth. High salinity has been reported as a stressful condition that affects the physiology, transcription, and membrane transport functions of EABf, which results in lower produced currents [[28], [29], [30]]. Other reasons that could explain the measured thin EABfs buffered with 100 mM are: 1) depletion of important nutrients for EABfs formation (for example calcium) due to their binding to phosphate, 2) an uneven disposition of the EABfs on the electrode that could lead to an underestimation of the EABf thickness measured with the OCT, and 3) changes in the microbial community. However, the continuous operation mode of all the reactors, the OCT scans, and the high obtained CE in this dataset exclude these other suggested reasons for the little growth of EABfs buffered with 100 mM.

Similar ranges of EABfs thicknesses have been reported in studies aiming at understating the effect of buffer on anodic EABfs. Yang et al. [31] measured final EABfs thicknesses of 42.6, 52.2 and 60.0 μm when phosphate buffer concentrations of 5, 50 and 100 mM were used, respectively. In this study, higher buffer concentration led to an increase in extracellular polymeric substances (EPS) production and steered the microbial community of the EABf towards a EABf dominated by *Geobacter* species. In another study with *Thermincola ferriacetica* and using bicarbonate buffer, Lusk et al., 2016 reported an increase in EABfs thickness from 68 μm with 10 mM buffer concentration to EABfs thicker than 150 μm with 100 mM buffer concentration. In both works, higher current densities and thicker EABfs were reported with increasing buffer concentrations, which could be related to the more positive anode potentials (−0.08 V and +0.14 V and vs Ag/AgCl) when compared to the anode potentials used in this study (−0.3 V and −0.2 V vs Ag/AgCl).

### 3.3 Maximum thickness of a non-buffer limited EABfs decreases with increasing anode potential

**Fig. 4:**
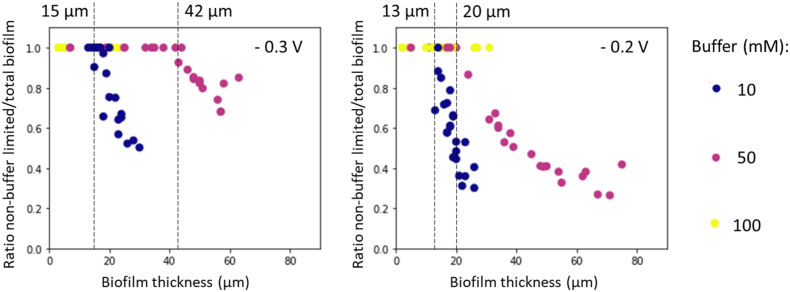
Ratio of non-buffer limited EABfs and total measured EABf on the electrode as a function of the EABf thickness measured with OCT for the three buffer concentrations (10 mM – blue; 50 mM purple, 100 mM – yellow), when the anode potential was controlled at −0.3 V (left) and −0.2 V (right) vs Ag/AgCl. (For interpretation of the references to colour in this figure legend, the reader is referred to the Web version of this article.)

Calculation of the penetration depths of acetate and buffer in EABfs enabled the determination of the ratio between the thickness of a non-limited EABfs and total measured EABf thickness. After comparing the acetate and buffer penetration depths with the measured EABf thickness with the OCT, the smallest dimension was chosen and divided by the measured EABf thickness. Thus, the ratio of non-limited EABf and total measure EABf thickness was determined, and it indicates the fraction of EABf that was not limited in acetate nor buffer and therefore, able to contribute to the produced current. Fig. 4 depicts this ratio as a function of the measured EABf thickness on the electrode for the three buffer concentrations tested at both anode potentials. As intended with the experimental plan, the use of non-limiting acetate concentrations resulted in the growth of EABfs that were only buffer limited (in other words, the acetate penetration depth was always bigger than the buffer penetration depth and the measured EABf thickness). Therefore, buffer limited EABfs were found when the calculated ratio was lower than 1, and when the ratio equaled 1, the buffer (and acetate) penetration depth was bigger than the measured EABf thickness. As an example, when the ratio of non-buffer limited EABfs equals 0.6, this means that 60% of the EABfs is non-buffer limited and the remaining 40% of the EABfs is buffer limited. Fig. 4 shows that buffer limitations occurred at both anode potentials when the EABfs were buffered with 10 and 50 mM buffer concentrations. Given the low range of EABfs thicknesses measured when the EABfs were buffered with 100 mM, no buffer limitations were found at this high buffer concentration (ratio of 1 for all EABfs as depicted in Fig. 4).

When the buffer concentration increased from 10 to 50 mM, the thickness of non-buffer limited EABfs increased from 15 μm to 42 μm at −0.3 V and from 13 μm to 20 μm at −0.2 V. Thus, the same increase in buffer concentration resulted in a smaller increase in the maximum thickness of a non-buffer limited EABf when a more positive anode potential was used. This indicates that the buffering capacity decreases with increasing anode potential. Besides, for a given buffer concentration, the maximum thickness of a non-buffer limited EABf decreased when a more positive anode potential was used. These results are related to the higher acetate consumption (and higher produced current) at more positive anode potentials (approximately 1.4 mM of acetate were consumed at −0.3 V and 1.7 mM of acetate were consumed at −0.2 V), which makes buffer the limiting factor for thinner EABfs. When more acetate is consumed, more buffer is used to neutralize the higher concentration of generated protons in the EABf and, therefore, lower available hydrogen phosphate remains present in the anolyte. Therefore, the buffering capacity decreases (lower buffer penetration depths), resulting in ratios lower than 1 at a thinner range of EABfs thicknesses. This is further emphasized when comparing the ratios between the thickness of a non-limited EABfs and total measured EABf thickness on the FTO electrode obtained at the two anode potentials: the lowest ratio at −0.3 V is approximately 0.5 when 10 mM buffer concentration was used and 0.7 when 50 mM buffer concentration was used, whereas ratios of approximately 0.2 were obtained at −0.2 V when both 10 and 50 mM buffer concentrations were used. This low ratio indicates that about 80% of the measured EABf thickness on the electrode was buffer limited.

### 3.4 Determining the boundary between acetate and buffer limited EABfs

Acetate and buffer concentration in the anolyte are two intertwined parameters that can be controlled to avoid limitations in EABfs on an anode. As previously shown, higher buffer capacities are needed when the anode is poised at more positive potentials, as more acetate is consumed. This indicates that the relation between the buffer concentration needed to avoid accumulation of protons in the EABf and the acetate concentration present in the anolyte depends on the anode potential.

By estimating both the acetate and buffer diffusion rates and specific acetate utilization rate of EABfs at two anode potentials (Table 1), the boundary between acetate and buffer limited EABfs can be determined.

**Table 1:** Acetate and buffer diffusion rates, and specific acetate utilization rate in EABfs when the anode potential was controlled at −0.3 V and −0.2 V vs Ag/AgCl.

Parameters	E = - 0.3 V	E = - 0.2 V
*k*_*0*_ (mol Ac.m^−3^.s^−1^)	0.05	0.08
*D*_*acetate*_ (m^2^.s^−1^)	2.22 × 10^−10^	2.20 × 10^−10^
*D*_*buffer*_ (m^2^.s^−1^)	3.33 × 10^−10^	1.31 × 10^−10^

The estimated specific acetate utilization rate increased from 0.05 to 0.08 mol Ac.m^−3^.s^−1^ when the anode potential increased from −0.3 to −0.2 V. The higher specific acetate utilization rate at −0.2 V confirms the higher acetate consumption and the higher buffer capacity needed at higher anode potentials. The model also estimated acetate diffusion rates of 2.22 × 10^−10^ m^2^ s^−1^ at −0.3 V and 2.20 × 10^−10^ m^2^ s^−1^ at −0.2 V, and buffer diffusion rates of 3.33 × 10^−10^ m^2^ s^−1^ at −0.3 V and 1.31 × 10^−10^ m^2^ s^−1^ at −0.2 V. The acetate diffusion rates are in the same order of magnitude of the acetate diffusion rates commonly used in modeling works with EABfs, however these are commonly calculated based on the diffusion rate of acetate and buffer in water and adjusted to diffusion rates in EABfs using empiric coefficients [17,19,[32], [33], [34]]. Given the identified buffer limitations in the EABfs studied in this work, the modeling approach was reproduced with the dataset previously reported by Pereira et al., 2022, only considering the EABfs that were not buffer limited (these EABfs were buffered with 50 mM, and no buffer limitations were identified, for both experiments at −0.2 and −0.3 V, when the acetate concentrations in the anolyte was lower than 6.6 mM). The estimated buffer diffusion rates with the non-buffer limited EABfs were very similar to the diffusion rates obtained in this study (2.47 × 10^−10^ m^2^ s^−1^ at −0.3 V and 3.19 × 10^−10^ m^2^ s^−1^ at −0.2 V), validating the use of the buffer diffusion rates here reported to calculate the boundaries between acetate and buffer limitations in EABfs.

**Fig. 5:**
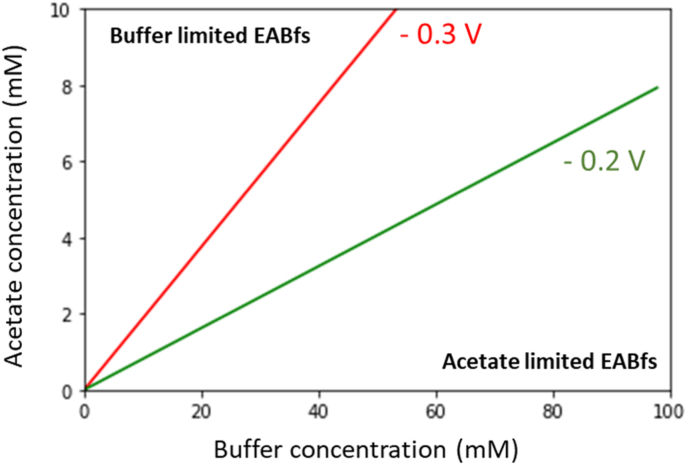
Definition of the boundaries between acetate and buffer limited EABfs when the anode potential is controlled at −0.3 V (red) and −0.2 V (green) vs Ag/AgCl. Combination of acetate and buffer concentrations above the boundary leads to buffer limited EABfs, and below the boundary leads to acetate limited EABfs. (For interpretation of the references to colour in this figure legend, the reader is referred to the Web version of this article.)

The boundary between acetate and buffer limitations in EABfs was calculated using Fick's law and the acetate and buffer diffusion rates estimated in this study. The acetate and buffer concentrations in the anolyte were derived from Fick's law considering a range of minimum penetration depths (*L*_minimum_) up to 100 μm. This range was chosen to result in an acetate concentration of 10 mM and a buffer concentration of 100 mM in the anolyte, matching the conditions used in the study. Fig. 5 shows the range of conditions under which EABfs are acetate and buffer limited when the anode potential is controlled at −0.2 and −0.3 V. The boundaries defined for −0.3 and −0.2 V divide the conditions under which EABfs are prompt to be buffer (above boundary) or acetate (below boundary) limited.

The slopes of the two boundaries indicate that the buffer concentration should be approximately 5 times higher than the acetate concentration when the anode is controlled at −0.3 V, and approximately 12 times when the anode potential is increased to −0.2 V. These ratios of buffer and acetate emphasize that, when acetate consumption is bio-catalyzed in an EABf, the stochiometric relation between acetate and protons (Eq. (1)) should also consider diffusion rates of acetate and buffer.

It is important to note that the defined boundaries only consider acetate and buffer limitations, leaving out EABfs thicknesses on the anode. Too little EABf growth on an electrode can also limit the current density. These “biomass” limited EABfs can occur throughout the whole range of acetate and buffer concentrations shown in the axis of Fig. 5 (with their highest frequency of occurrence expected to be located at the highest range of buffer and acetate concentrations) (Section E, Appendices). Besides their thickness, other parameters such as the composition of the extracellular polymeric matrix, cell density, and the morphology of EABfs are also very important as different EABfs structures and compositions could potentially change the acetate and buffer concentrations under which limitations occur. Nevertheless, in an industrial point of view, these boundaries are an important tool to control and to increase the energy recovered with these BESs, by showing the buffer concentration required as a function of the acetate concentration present in a real wastewater stream. Finally, both in a performance and in a research point of view, considering the diffusion of substrate and products in relation to EABf thickness could also bring valuable information to increase the performance of cathodic EABfs.

## 4 Conclusion

Real-time measurement of EABfs thicknesses is of key importance to understand how EABfs respond to provided operating conditions. As shown in this study, thicker EABfs do not always produce higher currents at the anode, as thick EABfs are more prompt to run into limitations. Therefore, determining the maximum thicknesses of non-limited EABfs, and controlling the thicknesses of EABfs on the anode up to the determined maximum thicknesses, is crucial to prevent limitations and to guarantee high produced currents. Moreover, identifying the limiting factor at a wide range of conditions is also a very important tool to control limitations towards higher produced currents.

## Funding

This work was supported by the “Resource Recovery” theme of Wetsus; and Dutch Research Council (10.13039/501100003246NWO) [project “Understanding and controlling electron flows in electro-active biofilms” with project number 17516 that is part of the research program Vidi].

## CRediT authorship contribution statement

**João Pereira:** Conceptualization, Methodology, Investigation, Writing – original draft. **Guanxiong Wang:** Methodology, Validation. **Tom Sleutels:** Conceptualization, Writing – review & editing. **Bert Hamelers:** Conceptualization, Writing – review & editing. **Annemiek ter Heijne:** Conceptualization, Writing – review & editing, Supervision, Project administration, Funding acquisition.

## Declaration of competing interest

The authors declare that they have no known competing financial interests or personal relationships that could have appeared to influence the work reported in this paper.

## Data Availability

doi: 10.4121/20268213
