# Supplementary material for: Maximum thickness of non-buffer limited electro-active biofilms decreases at higher anode potentials
Source: Biofilm. 2022 Nov 11;4:100092. doi: 10.1016/j.bioflm.2022.100092 (PMC9678801; doi:10.1016/j.bioflm.2022.100092)
Supplement: Multimedia component 1 [file mmc1.docx]

**Appendices**

1. **High Coulombic efficiencies proving the presence of a EABfs on the anode**

To calculate the Coulombic efficiency of the EABfs, acetate concentration in the anolyte was calculated based on the produced current. Equation A1 was used to calculate the acetate concentration in the anolyte, *Ac_out,equi_* (mM acetate), in which *Ac_in_* is the measured acetate concentration in the influent (mM acetate), *i* is the measured current (C.s^-1^), *flow* (0.16 mL.min^-1^) is the flowrate, *F* is the Faraday constant (96485 Coulombs.mol_electrons_^-1^) and *n* (8 mol_electrons_.mol_acetate_^-1^) is the number of moles of electrons produced per mole of acetate consumed.

*Ac_out,equi_* = *Ac_in_* – *i*/*flow*/(*F*×*n*) (Eq. A1)

The Coulombic efficiency (CE) can be determined by the ratio between the calculated concentration of acetate in the anolyte based on current produced, *Ac_out,equi_* (meaning the difference between the acetate concentration in the influent and the consumed acetate based on produced current) and the measured acetate concentration in the anolyte, *Ac_out_* (Equation A2). Figure A1 shows that the Coulombic efficiency was close to 100% in all experiments.

CE = *Ac_out,equi_* /*Ac_out_* (Eq. A2)
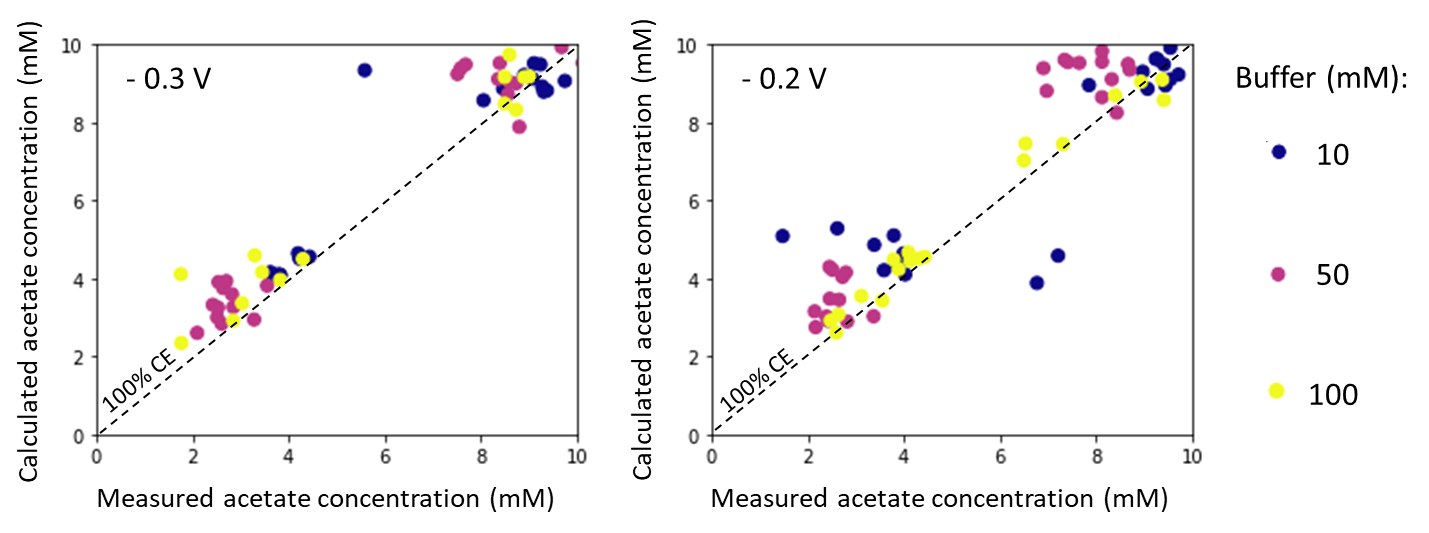


**Figure A1**. Relation between measured and calculated acetate concentration in the anolyte for the three buffer concentrations (10 mM – blue; 50 mM purple, 100 mM – yellow), when the anode potential was controlled at -0.3 V (left) and -0.2 V (right) vs Ag/AgCl.

Therefore, competitive processes in the anode such as the presence of methanogens and sulphur reducers are neglected, making this experimental setup suitable to study EABfs.

1. **Script used for the modelling approach**

Python was used to quantify buffer and acetate penetration depths and to estimate buffer and acetate diffusion rate and specific acetate utilization rates in EABfs. Some lines of the script used to translate the equations mentioned in the Material and Method section into Python language are discriminated below. The estimated specific acetate utilization rate, acetate diffusion and buffer diffusion are coded as par[0], par[1], and par[2], respectively.

*“data_pd[HPO_4_^2-^ concentration']=10**(data_pd['anolyte pH]-7.2)/(1+10**(data_pd['anolyte pH']-7.2))*data_pd['Buffer concentration (mM)']/8*

*data_pd['Buffer penetration depth'] = (2*np.abs(par[2])*data_pd[' HPO_4_^2-^ concentration'']).apply(np.sqrt)*

*data_pd['Acetate penetration depth'] = (2*np.abs(par[1])*data_pd['Acetate concentration (mM)']).apply(np.sqrt)*

*data_pd['L_minimum_']=(data_pd[['Buffer penetration depth','Biofilm thickness (µm)', 'Acetate penetration depth']].min(axis=1))*

*data_pd['Estimated current density']=data_pd['L_minimum_']*par[0]*

*data_pd['Ratio non-buffer limited/total biofilm']=data_pd[' L_minimum_ ']/data_pd['Biofilm thickness (µm)']*

*data_pd['calculation of squares']=(data_pd['Measured current density']-data_pd['Estimated current density'])**2”*

*return data_pd['calculation of squares'].sum()”*

Nelder-Mead was used as the method to minimize the variation between estimated and measured current. Equation B1 was used to calculate the “*correlation factor*”, in which *ssq mode* is the mode of the sum of squares and *ssq avg* is the average of the sum of squares. A correlation of approximately 0.4 was obtained for each group of data divided by the applied anode potential.

*correlation factor* = 1 - *ssq mode*/*ssq avg* (Eq. B1)

1. **Determining the anolyte pH based on produced current in a non-buffered anode**

Eq. C1 shows the calculation of the anolyte pH based on the current produced (electrons produced from acetate oxidation, which equals the number of protons produced) in case no buffer was present in the influent. *pH_no buffer_* is the expected anolyte pH in the absence of buffer in the influent, *j* is the measured current density (A.m^-2^), *electrode area* is the operating area of the electrode (0.0023 m^2^), *flow* is the flowrate (0.16 mL.min^-1^ x 0.001 L.mL^-1^ x 1/60 min.s^-1^) and *F* is the faraday constant (96485 96485 Coulombs.mol_electrons_^-1^).

*pH_no buffer_* = -log_10_ (*j* x *electrode area*/*flow*/*F*) (Eq. C1)

1. **The effect of the buffer concentration on the produced current profiles**

Figure D1 shows the produced current profile and the EABfs growth for all the experiments performed in this study. We observed an initial increase in produced current that was followed by a constant and stable current produced over time (Figure D1.a). For experiments with 100 mM buffer concentration, the produced current decreased after the peak and stabilized at a value lower than the peak. The amount of biomass present on the electrode is shown in Figure D1.b. Even though current profiles were different for each experiment, an increase in biomass with time was observed for the period evaluated in all experiments. This shows that while current was produced, even when current decreased, biomass thickness kept increasing on the FTO electrode.


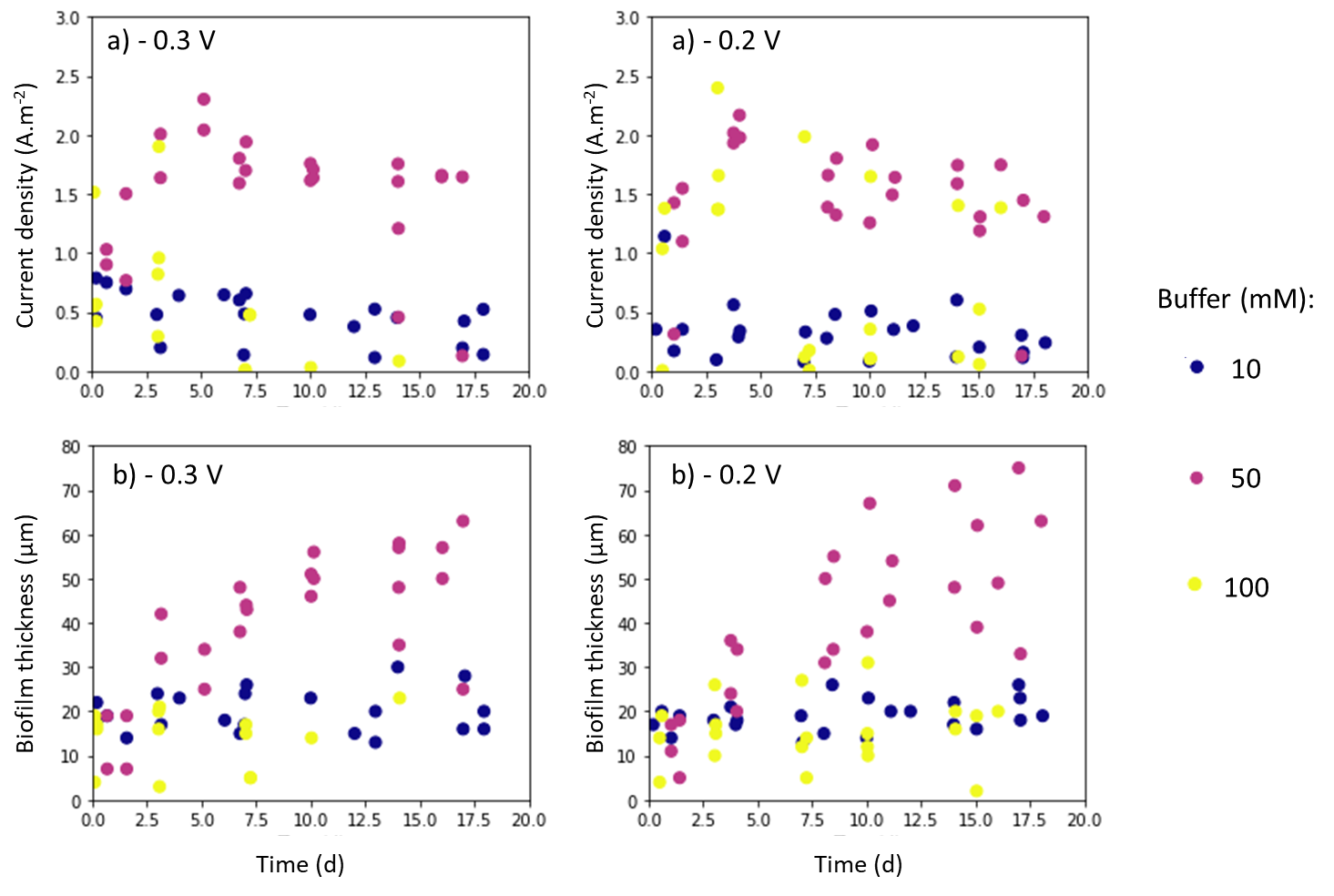


**Figure D1.** a) Current density and b) measured EABf thickness on the FTO electro as a function of time for the three buffer concentrations (10 mM – blue; 50 mM purple, 100 mM – yellow), when the anode potential was controlled at -0.3 V (left) and -0.2 V (right) vs Ag/AgCl.

1. **Determining the boundary between acetate and buffer limited EABfs**

Figure E1 shows the boundaries between buffer and acetate limited EABfs and the distribution of the experimental data. In this Figure, “biomass” limited EABfs obtained in the experiments performed in this study are also discriminated (in purple).


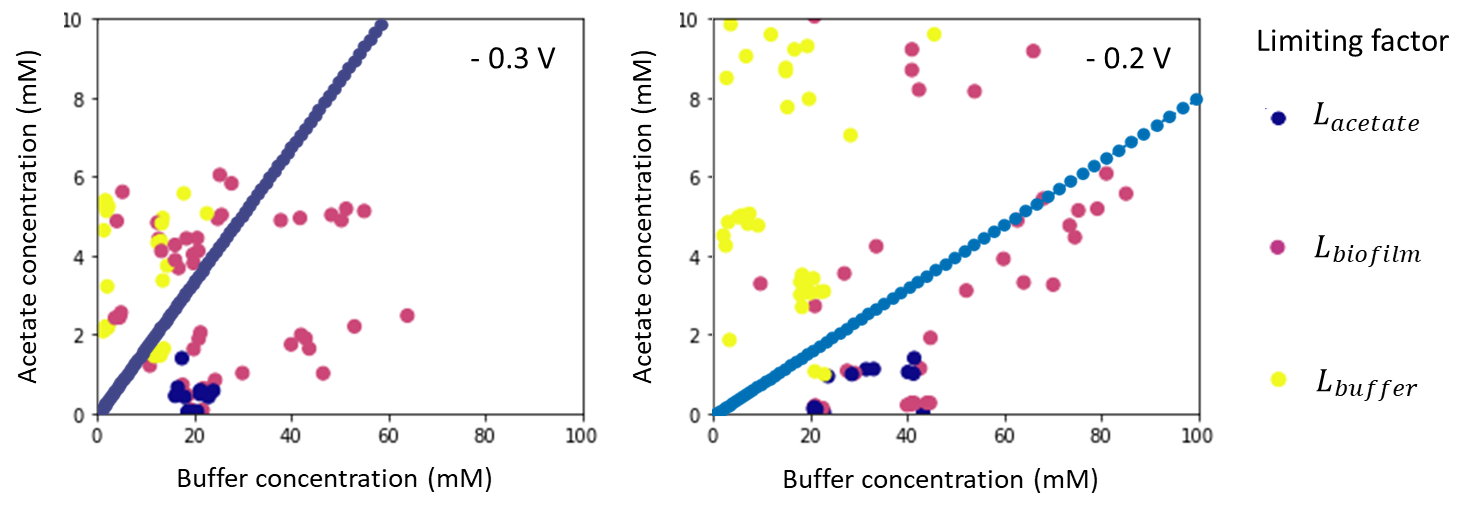


**Figure E1.** Boundary between acetate and buffer limited EABfs and distribution of the experimental data when the anode potential was controlled at -0.3 V (left) and -0.2 V (right) vs Ag/AgCl. Acetate limited EABfs are colored in blue, buffer limited EABfs are colored in yellow and “biomass” limited EABfs are colored in purple.
